# 7,9-Dichloro-6*H*,12*H*-indolo[2,1-*b*]quinazoline-6,12-dione

**DOI:** 10.1107/S1600536810018969

**Published:** 2010-05-29

**Authors:** Peter Grundt, Kelsi A. Douglas, Bogdana Krivogorsky, Victor N. Nemykin

**Affiliations:** a Department of Chemistry & Biochemistry, University of Minnesota Duluth, 1039 University Drive, Duluth, MN 55812, USA

## Abstract

There are two independent mol­ecules in the asymmetric unit of the title compound, C_15_H_6_Cl_2_N_2_O_2_. The conjugated four-ring system is essentially planar in each mol­ecule [maximum deviation = 0.089 (2) Å]. In the crystal, weak inter­molecular C—H⋯Cl, C—H⋯O and C—H⋯·N inter­actions help to stabilize the packing.

## Related literature

For the synthesis, chemistry, and biological activity of the title compound see: Krivogorsky *et al.* (2008 ▶). For chemistry and biological activity of the natural product tryptanthrin (indolo[2,1-*b*]quinazoline-6,12-dione) and its derivatives and for related structures, see: Honda *et al.* (1979 ▶); Mitscher & Baker (1998 ▶); Kataoka *et al.* (2001 ▶); Bandekar *et al.* (2010 ▶); Sharma *et al.* (2002 ▶); Motoki *et al.* (2005 ▶); Yu *et al.* (2009 ▶); Bhattacharjee *et al.* (2002 ▶); Scovill *et al.* (2002 ▶); Bhattacharjee *et al.* (2004 ▶); Pitzer *et al.* (2000 ▶). For the extinction correction, see: Larson (1970 ▶).
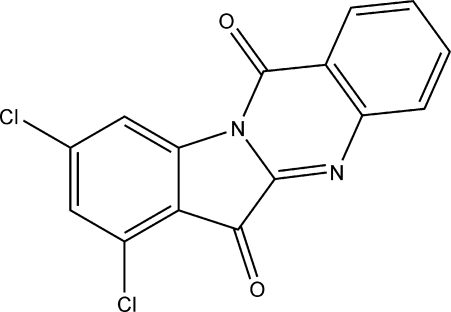


## Experimental

### 

#### Crystal data

C_15_H_6_Cl_2_N_2_O_2_
*M* *_r_* = 317.13
Triclinic, 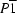

*a* = 7.0179 (2) Å
*b* = 10.7276 (3) Å
*c* = 17.2338 (12) Å
α = 94.908 (7)°
β = 96.709 (7)°
γ = 107.395 (8)°
*V* = 1219.66 (12) Å^3^
*Z* = 4
Mo *K*α radiation
μ = 0.54 mm^−1^
*T* = 100 K
0.54 × 0.48 × 0.35 mm

#### Data collection

Rigaku R-AXIS RAPID-II imaging plate diffractometer
Absorption correction: multi-scan (*ABSCOR*; Higashi, 1995 ▶) *T*_min_ = 0.633, *T*_max_ = 0.899
31502 measured reflections
5585 independent reflections
4830 reflections with *I* > 2σ(*I*)
*R*_int_ = 0.049

#### Refinement

*R*[*F* ^2^ > 2σ(*F* ^2^)] = 0.034
*wR*(*F* ^2^) = 0.082
*S* = 1.00
5571 reflections
416 parameters
84 restraints
All H-atom parameters refined
Δρ_max_ = 0.53 e Å^−3^
Δρ_min_ = −0.36 e Å^−3^

### 

Data collection: *CrystalClear* (Rigaku Americas, 2009 ▶); cell refinement: *HKL-2000* (Otwinowski & Minor, 1997 ▶); data reduction: *CrystalClear*; program(s) used to solve structure: *SHELXS86* (Sheldrick, 2008 ▶); program(s) used to refine structure: *CRYSTALS* (Betteridge *et al.*, 2003 ▶); molecular graphics: *CAMERON* (Watkin *et al.*, 1996 ▶); software used to prepare material for publication: *CRYSTALS*.

## Supplementary Material

Crystal structure: contains datablocks global, I. DOI: 10.1107/S1600536810018969/jj2032sup1.cif

Structure factors: contains datablocks I. DOI: 10.1107/S1600536810018969/jj2032Isup2.hkl

Additional supplementary materials:  crystallographic information; 3D view; checkCIF report

## Figures and Tables

**Table 1:** Hydrogen-bond geometry (Å, °)

*D*—H⋯*A*	*D*—H	H⋯*A*	*D*⋯*A*	*D*—H⋯*A*
C1*B*—H1*B*⋯Cl1*A*^i^	0.94 (2)	2.73 (2)	3.637 (2)	162 (1)
C2*A*—H2*A*⋯O1*B*^ii^	0.93 (2)	2.54 (2)	3.264 (3)	135 (1)
C4*B*—H4*B*⋯N5*A*^iii^	0.94 (2)	2.56 (2)	3.422 (3)	154 (1)
C10*A*—H6*A*⋯Cl2*B*^iv^	0.94 (2)	2.67 (2)	3.585 (2)	165 (1)

Symmetry codes: (i) 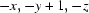; (ii) 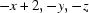; (iii) 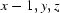; (iv) 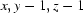.

**Fig. 1.**
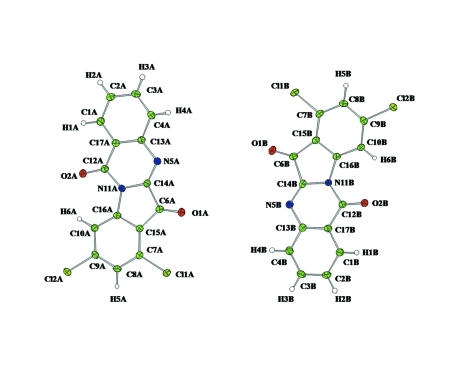
The title compound with displacement ellipsoids drawn at the 50% probability level. H atoms are shown as spheres of arbitary radius.

**Fig. 2.**
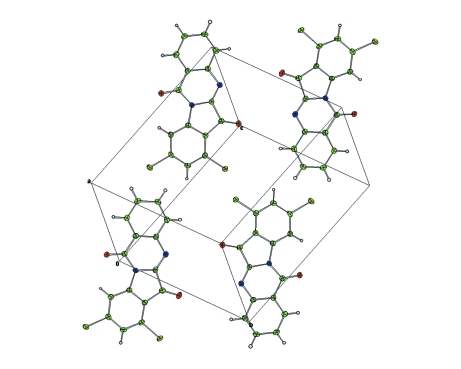
Packing diagram for the title compound with displacement ellipsoids drawn at the 50% probability level. H atoms are omited for clarity.

## supplementary crystallographic information

### Comment

The natural product tryptanthrin (indolo[2,1-b]quinazoline-6,12-dione) and its derivatives have been shown to possess antibacterial (Honda *et al.*, 1979), Mitscher & Baker, 1998, Kataoka *et al.*, 2001, Bandekar *et al.*, 2010) and antitumor Sharma *et al.*, 2002, Motoki *et al.*, 2005, Yu *et al.*, 2009) properties. Of particular interest is the discovery by several groups that this class of compounds also inibits the growth of parasites such as *Leishmania donovani* (Bhattacharjee *et al.*, 2002), *Trypanosoma brucei* (Scovill *et al.*, 2002), and *Plasmodium falciparum* (Bhattacharjee *et al.*, 2004, Pitzer *et al.*, 2000), and more recently by our laboratory, *Toxoplasma gondii* (Krivogorsky *et al.*, 2008). In our continued interest to characterize the structure-activity-relationship of this class of compounds and to reveal the underlying mechanism, we have synthesized the 7,9-dichloro analog of tryptanthrin.

The title compound, (I), C_15_H_6_Cl_2_N_2_O_2_, crystallizes in the P-1 space group with two independent molecules in the asymmetric unit cell.  It consists of a 7,9-dichloroindolo ring fused to a quinazoline ring with a dione group at the 6 and 12 poisitions (IUPAC nomenclature). C—Cl bond distances have been observed between 1.7272 (19) and 1.7358 (19) Å with Cl1—C7 distances being slightly shorter as compared to Cl2—C9 bond lengths. C=O bonds have clear double bond character and were observed between 1.211 (2) and 1.221 (2) Å with C=O bonds in the five-membered ring being slightly shorter as compared to those at the six-membered rings. N5—C14 bond distances in molecules A and B have clear double bond character. Four weak intermolecular interactions are observed in (I), (Table 2) that help stabilize crystal packing.

### Experimental

The title compound was prepared by condensation of isatoic anhydride and 4,6-dichloroisatin in refluxing benzene with triethylamine as a co-solvent (Krivogorsky *et al.*, 2008). Crystals suitable for single-crystal X-ray diffraction were grown by slow evaporation of an acetone solution of the compound.

### Refinement

In the absence of significant anomalous scattering, Friedel pairs were merged. The H atoms were all located in a difference map, but were repositioned geometrically. The H atoms were initially refined with soft restraints on the bond lengths and angles to regularize their geometry (C—H in the range 0.93–0.94 Å) and *U*_iso_(H) (in the range 1.2–1.5 times *U*_eq_ of the parent atom), after which the positions were refined with riding constraints.

### Crystal data

**Table:**

C_15_H_6_Cl_2_N_2_O_2_	*Z* = 4
*M**_r_* = 317.13	*F*(000) = 640
Triclinic, *P*1	*D*_x_ = 1.727 Mg m^−^^3^
Hall symbol: -P 1	Melting point: 200 K
*a* = 7.0179 (2) Å	Mo *K*α radiation, λ = 0.71075 Å
*b* = 10.7276 (3) Å	Cell parameters from 28356 reflections
*c* = 17.2338 (12) Å	θ = 3.1–27.5°
α = 94.908 (7)°	µ = 0.54 mm^−^^1^
β = 96.709 (7)°	*T* = 100 K
γ = 107.395 (8)°	Block, yellow
*V* = 1219.66 (12) Å^3^	0.54 × 0.48 × 0.35 mm

### Data collection

**Table:**

Rigaku R-AXIS RAPID-II imaging plate diffractometer	5585 independent reflections
Radiation source: Sealed tube (Mo)	4830 reflections with *I* > 2σ(*I*)
graphite	*R*_int_ = 0.049
Detector resolution: 10 pixels mm^-1^	θ_max_ = 27.5°, θ_min_ = 3.1°
ω scans	*h* = −9→9
Absorption correction: multi-scan (*ABSCOR*; Higashi, 1995)	*k* = −13→13
*T*_min_ = 0.633, *T*_max_ = 0.899	*l* = −22→22
31502 measured reflections	

### Refinement

**Table:**

Refinement on *F*^2^	Secondary atom site location: difference Fourier map
Least-squares matrix: full	Hydrogen site location: inferred from neighbouring sites
*R*[*F*^2^ > 2σ(*F*^2^)] = 0.034	All H-atom parameters refined
*wR*(*F*^2^) = 0.082	Method = Modified Sheldrick *w* = 1/[σ^2^(*F*^2^) + (0.02*P*)^2^ + 2.09*P*], where *P* = [max(*F*_o_^2^,0) + 2*F*_c_^2^]/3
*S* = 1.00	(Δ/σ)_max_ = 0.001
5571 reflections	Δρ_max_ = 0.53 e Å^−^^3^
416 parameters	Δρ_min_ = −0.36 e Å^−^^3^
84 restraints	Extinction correction: Larson (1970), Equation 22
0 constraints	Extinction coefficient: 43 (4)
Primary atom site location: structure-invariant direct methods	

### Special details

**Table:**

Experimental. The crystal was placed in the cold stream of an X-stream 2000 liquid nitrogen generator with open-flow nitrogen cryostat with a nominal stability of 0.1 K. ^1^H NMR (DMSO-d6, 500 MHz): d 7.75-7.78 (m, 2H), 7.98-8.00 (m, 2H), 8.33 (d, J 7.5, 1H), 8.41 (d, J 1.9, 1H). ^13^C NMR (DMSO-d6, 125 MHz): d 115.6, 118.3, 122.6, 127.0, 127.4, 129.9, 130.1, 132.3, 135.6, 141.6, 144.4, 146.1, 147.3, 157.6, 178.4.
Refinement. Crystals for Windows program eliminates all reflections with [Sin theta/lambda]**2 less than 0.01 in order to eliminate reflections that may be poorly measured in the vicinity of the beam stop. Such filter eliminated 14 reflections, which resulted in difference between 5585 measured unique reflections and 5571 reflections used for refinement.

### Fractional atomic coordinates and isotropic or equivalent isotropic displacement parameters (Å^2^)

**Table:**

	*x*	*y*	*z*	*U*_iso_*/*U*_eq_	
Cl1A	0.12767 (7)	0.19849 (5)	−0.30745 (3)	0.0154	
Cl2A	0.22470 (8)	−0.16786 (5)	−0.51632 (3)	0.0203	
O1A	0.5092 (2)	0.25603 (14)	−0.16905 (8)	0.0172	
O2A	0.8111 (2)	−0.15104 (14)	−0.30107 (8)	0.0181	
C1A	1.1319 (3)	−0.08669 (19)	−0.17069 (12)	0.0162	
C2A	1.2819 (3)	−0.0485 (2)	−0.10617 (12)	0.0183	
C3A	1.2850 (3)	0.0523 (2)	−0.04843 (12)	0.0208	
C4A	1.1391 (3)	0.1145 (2)	−0.05462 (12)	0.0185	
N5A	0.8403 (2)	0.14280 (16)	−0.12490 (10)	0.0151	
C6A	0.5402 (3)	0.16629 (19)	−0.20790 (11)	0.0137	
C7A	0.2640 (3)	0.09548 (18)	−0.33341 (11)	0.0137	
C8A	0.1974 (3)	0.01396 (19)	−0.40464 (11)	0.0154	
C9A	0.3055 (3)	−0.06961 (19)	−0.42595 (11)	0.0151	
C10A	0.4729 (3)	−0.07980 (19)	−0.37831 (11)	0.0146	
N11A	0.6952 (2)	0.01079 (16)	−0.24828 (9)	0.0130	
C12A	0.8273 (3)	−0.06404 (19)	−0.24787 (11)	0.0140	
C13A	0.9855 (3)	0.07665 (19)	−0.11960 (11)	0.0142	
C14A	0.7090 (3)	0.10816 (18)	−0.18691 (11)	0.0134	
C15A	0.4331 (3)	0.09159 (18)	−0.28464 (11)	0.0129	
C16A	0.5321 (3)	0.00196 (19)	−0.30772 (11)	0.0141	
C17A	0.9828 (3)	−0.02472 (18)	−0.17809 (11)	0.0139	
Cl1B	0.89170 (7)	0.34858 (5)	0.34019 (3)	0.0173	
Cl2B	0.70190 (7)	0.70635 (5)	0.52404 (3)	0.0192	
O1B	0.5337 (2)	0.26130 (14)	0.19080 (9)	0.0189	
O2B	0.1512 (2)	0.64871 (14)	0.28391 (8)	0.0169	
C1B	−0.1458 (3)	0.55847 (19)	0.14382 (12)	0.0163	
C2B	−0.2819 (3)	0.5080 (2)	0.07583 (12)	0.0193	
C3B	−0.2635 (3)	0.4038 (2)	0.02547 (12)	0.0192	
C4B	−0.1086 (3)	0.3521 (2)	0.04302 (12)	0.0176	
N5B	0.1872 (3)	0.34718 (16)	0.12667 (10)	0.0153	
C6B	0.4831 (3)	0.34864 (19)	0.22288 (11)	0.0148	
C7B	0.7333 (3)	0.44170 (19)	0.35489 (12)	0.0146	
C8B	0.7737 (3)	0.5271 (2)	0.42418 (12)	0.0165	
C9B	0.6463 (3)	0.60188 (19)	0.43630 (11)	0.0151	
C10B	0.4808 (3)	0.59806 (19)	0.38219 (11)	0.0144	
N11B	0.2930 (2)	0.49043 (16)	0.24801 (9)	0.0130	
C12B	0.1523 (3)	0.55777 (19)	0.23644 (11)	0.0138	
C13B	0.0312 (3)	0.40238 (18)	0.11173 (11)	0.0139	
C14B	0.3049 (3)	0.39217 (19)	0.19191 (11)	0.0140	
C15B	0.5689 (3)	0.43274 (19)	0.29911 (11)	0.0139	
C16B	0.4478 (3)	0.51304 (19)	0.31342 (11)	0.0135	
C17B	0.0111 (3)	0.50602 (19)	0.16292 (11)	0.0141	
H1A	1.130 (2)	−0.1539 (13)	−0.2094 (9)	0.0204*	
H2A	1.380 (2)	−0.0909 (13)	−0.1021 (9)	0.0206*	
H3A	1.388 (2)	0.0793 (14)	−0.0051 (9)	0.0251*	
H4A	1.142 (2)	0.1815 (13)	−0.0153 (9)	0.0215*	
H5A	0.083 (2)	0.0145 (13)	−0.4368 (9)	0.0178*	
H6A	0.541 (2)	−0.1385 (13)	−0.3938 (9)	0.0187*	
H1B	−0.159 (2)	0.6286 (13)	0.1773 (9)	0.0204*	
H2B	−0.388 (2)	0.5439 (14)	0.0634 (9)	0.0248*	
H3B	−0.355 (2)	0.3688 (14)	−0.0209 (9)	0.0224*	
H4B	−0.094 (2)	0.2847 (13)	0.0083 (9)	0.0208*	
H5B	0.885 (2)	0.5353 (13)	0.4615 (9)	0.0195*	
H6B	0.398 (2)	0.6491 (12)	0.3915 (9)	0.0160*	

### Atomic displacement parameters (Å^2^)

**Table:**

	*U*^11^	*U*^22^	*U*^33^	*U*^12^	*U*^13^	*U*^23^
Cl1A	0.0147 (2)	0.0165 (2)	0.0164 (2)	0.00768 (18)	0.00158 (17)	0.00171 (18)
Cl2A	0.0192 (2)	0.0251 (3)	0.0143 (2)	0.0077 (2)	−0.00299 (18)	−0.00561 (19)
O1A	0.0188 (7)	0.0163 (7)	0.0172 (7)	0.0078 (6)	0.0020 (6)	−0.0021 (6)
O2A	0.0182 (7)	0.0184 (7)	0.0177 (7)	0.0084 (6)	0.0001 (6)	−0.0036 (6)
C1A	0.0174 (10)	0.0143 (9)	0.0181 (10)	0.0060 (8)	0.0036 (8)	0.0031 (8)
C2A	0.0166 (10)	0.0196 (10)	0.0207 (10)	0.0089 (8)	0.0013 (8)	0.0042 (8)
C3A	0.0202 (10)	0.0255 (11)	0.0166 (10)	0.0097 (9)	−0.0037 (8)	0.0014 (8)
C4A	0.0196 (10)	0.0208 (10)	0.0138 (9)	0.0067 (8)	−0.0007 (8)	−0.0015 (8)
N5A	0.0154 (8)	0.0161 (8)	0.0138 (8)	0.0061 (7)	0.0005 (6)	0.0000 (6)
C6A	0.0131 (9)	0.0135 (9)	0.0146 (9)	0.0039 (7)	0.0020 (7)	0.0031 (7)
C7A	0.0137 (9)	0.0125 (9)	0.0154 (9)	0.0042 (7)	0.0033 (7)	0.0026 (7)
C8A	0.0122 (9)	0.0187 (10)	0.0144 (9)	0.0048 (8)	−0.0011 (7)	0.0024 (8)
C9A	0.0150 (9)	0.0151 (9)	0.0121 (9)	0.0016 (8)	0.0004 (7)	−0.0015 (7)
C10A	0.0134 (9)	0.0133 (9)	0.0162 (9)	0.0039 (7)	0.0012 (7)	−0.0005 (7)
N11A	0.0126 (8)	0.0131 (8)	0.0122 (8)	0.0038 (6)	−0.0005 (6)	−0.0011 (6)
C12A	0.0120 (9)	0.0138 (9)	0.0156 (9)	0.0025 (7)	0.0027 (7)	0.0024 (7)
C13A	0.0140 (9)	0.0137 (9)	0.0150 (9)	0.0043 (7)	0.0021 (7)	0.0030 (7)
C14A	0.0145 (9)	0.0116 (9)	0.0147 (9)	0.0046 (7)	0.0028 (7)	0.0010 (7)
C15A	0.0124 (9)	0.0121 (9)	0.0138 (9)	0.0028 (7)	0.0027 (7)	0.0024 (7)
C16A	0.0125 (9)	0.0145 (9)	0.0147 (9)	0.0033 (7)	0.0013 (7)	0.0027 (7)
C17A	0.0133 (9)	0.0130 (9)	0.0148 (9)	0.0034 (7)	0.0014 (7)	0.0028 (7)
Cl1B	0.0148 (2)	0.0186 (2)	0.0217 (2)	0.00967 (18)	0.00275 (18)	0.00399 (19)
Cl2B	0.0183 (2)	0.0231 (3)	0.0153 (2)	0.00847 (19)	−0.00197 (18)	−0.00346 (19)
O1B	0.0201 (7)	0.0182 (7)	0.0213 (7)	0.0103 (6)	0.0049 (6)	0.0004 (6)
O2B	0.0170 (7)	0.0174 (7)	0.0167 (7)	0.0085 (6)	−0.0007 (5)	−0.0024 (6)
C1B	0.0173 (9)	0.0151 (9)	0.0173 (9)	0.0070 (8)	0.0013 (8)	0.0010 (8)
C2B	0.0168 (10)	0.0214 (10)	0.0210 (10)	0.0097 (8)	−0.0012 (8)	0.0028 (8)
C3B	0.0179 (10)	0.0207 (10)	0.0162 (10)	0.0052 (8)	−0.0038 (8)	−0.0006 (8)
C4B	0.0207 (10)	0.0163 (9)	0.0157 (9)	0.0068 (8)	0.0010 (8)	−0.0003 (8)
N5B	0.0162 (8)	0.0149 (8)	0.0153 (8)	0.0059 (7)	0.0022 (6)	0.0011 (7)
C6B	0.0120 (9)	0.0157 (9)	0.0165 (9)	0.0035 (7)	0.0031 (7)	0.0038 (8)
C7B	0.0126 (9)	0.0141 (9)	0.0197 (10)	0.0065 (7)	0.0045 (7)	0.0048 (8)
C8B	0.0148 (9)	0.0190 (10)	0.0156 (9)	0.0057 (8)	−0.0010 (8)	0.0039 (8)
C9B	0.0160 (9)	0.0143 (9)	0.0128 (9)	0.0024 (7)	0.0013 (7)	−0.0001 (7)
C10B	0.0123 (9)	0.0158 (9)	0.0157 (9)	0.0055 (7)	0.0022 (7)	0.0009 (8)
N11B	0.0117 (7)	0.0142 (8)	0.0128 (8)	0.0048 (6)	0.0002 (6)	−0.0001 (6)
C12B	0.0120 (9)	0.0141 (9)	0.0155 (9)	0.0039 (7)	0.0022 (7)	0.0027 (7)
C13B	0.0145 (9)	0.0117 (9)	0.0158 (9)	0.0035 (7)	0.0030 (7)	0.0034 (7)
C14B	0.0141 (9)	0.0126 (9)	0.0166 (9)	0.0053 (7)	0.0049 (7)	0.0022 (7)
C15B	0.0124 (9)	0.0137 (9)	0.0162 (9)	0.0045 (7)	0.0034 (7)	0.0030 (7)
C16B	0.0106 (8)	0.0144 (9)	0.0152 (9)	0.0034 (7)	0.0009 (7)	0.0029 (7)
C17B	0.0145 (9)	0.0137 (9)	0.0144 (9)	0.0045 (7)	0.0024 (7)	0.0034 (7)

### Geometric parameters (Å, °)

**Table:**

Cl1A—C7A	1.7272 (19)	C1A—C2A	1.379 (3)
Cl2A—C9A	1.7358 (19)	C1A—H1A	0.937 (15)
Cl1B—C7B	1.7276 (19)	C2A—C3A	1.399 (3)
Cl2B—C9B	1.734 (2)	C2A—H2A	0.929 (16)
O1A—C6A	1.213 (2)	C3A—C4A	1.379 (3)
O2A—C12A	1.221 (2)	C3A—H3A	0.937 (16)
O1B—C6B	1.211 (2)	C4A—C13A	1.399 (3)
O2B—C12B	1.221 (2)	C4A—H4A	0.937 (15)
N5A—C13A	1.405 (2)	C14A—C6A	1.518 (3)
N5A—C14A	1.275 (2)	C15B—C7B	1.387 (3)
N11A—C16A	1.419 (2)	C15B—C16B	1.405 (3)
N11A—C12A	1.396 (2)	C15B—C6B	1.480 (3)
N11A—C14A	1.395 (2)	C7B—C8B	1.387 (3)
N5B—C13B	1.401 (2)	C8B—C9B	1.389 (3)
N5B—C14B	1.277 (3)	C8B—H5B	0.931 (15)
N11B—C16B	1.421 (2)	C9B—C10B	1.390 (3)
N11B—C12B	1.394 (2)	C10B—C16B	1.384 (3)
N11B—C14B	1.396 (2)	C10B—H6B	0.930 (15)
C15A—C7A	1.386 (3)	C12B—C17B	1.467 (3)
C15A—C16A	1.401 (3)	C17B—C1B	1.399 (3)
C15A—C6A	1.477 (3)	C17B—C13B	1.410 (3)
C7A—C8A	1.389 (3)	C1B—C2B	1.378 (3)
C8A—C9A	1.389 (3)	C1B—H1B	0.942 (15)
C8A—H5A	0.925 (15)	C2B—C3B	1.402 (3)
C9A—C10A	1.390 (3)	C2B—H2B	0.947 (16)
C10A—C16A	1.380 (3)	C3B—C4B	1.377 (3)
C10A—H6A	0.938 (15)	C3B—H3B	0.937 (16)
C12A—C17A	1.465 (3)	C4B—C13B	1.399 (3)
C17A—C1A	1.397 (3)	C4B—H4B	0.936 (16)
C17A—C13A	1.412 (3)	C14B—C6B	1.517 (3)
			
Cl1A—C7A—C15A	121.65 (15)	Cl1B—C7B—C15B	121.43 (15)
Cl1A—C7A—C8A	118.05 (15)	Cl1B—C7B—C8B	118.59 (15)
C15A—C7A—C8A	120.30 (18)	C15B—C7B—C8B	119.98 (18)
C7A—C15A—C6A	132.59 (18)	C7B—C15B—C6B	132.47 (18)
C7A—C15A—C16A	118.79 (17)	C7B—C15B—C16B	118.96 (18)
C6A—C15A—C16A	108.62 (16)	C6B—C15B—C16B	108.54 (16)
C15A—C6A—O1A	129.87 (18)	C15B—C6B—O1B	130.04 (18)
C15A—C6A—C14A	104.37 (15)	C15B—C6B—C14B	104.47 (16)
O1A—C6A—C14A	125.75 (17)	O1B—C6B—C14B	125.46 (18)
C6A—C14A—N5A	126.26 (17)	C6B—C14B—N11B	107.35 (16)
C6A—C14A—N11A	107.38 (16)	C6B—C14B—N5B	126.40 (17)
N5A—C14A—N11A	126.35 (17)	N11B—C14B—N5B	126.25 (17)
C14A—N5A—C13A	115.81 (17)	C14B—N11B—C16B	110.20 (15)
N5A—C13A—C4A	118.48 (17)	C14B—N11B—C12B	122.59 (16)
N5A—C13A—C17A	122.13 (17)	C16B—N11B—C12B	127.14 (16)
C4A—C13A—C17A	119.38 (18)	N11B—C16B—C15B	109.35 (16)
C13A—C4A—C3A	119.69 (19)	N11B—C16B—C10B	127.71 (17)
C13A—C4A—H4A	120.2 (10)	C15B—C16B—C10B	122.94 (18)
C3A—C4A—H4A	120.2 (10)	C16B—C10B—C9B	115.54 (18)
C4A—C3A—C2A	120.96 (19)	C16B—C10B—H6B	122.2 (10)
C4A—C3A—H3A	119.0 (10)	C9B—C10B—H6B	122.3 (10)
C2A—C3A—H3A	120.0 (10)	C10B—C9B—C8B	123.79 (18)
C3A—C2A—C1A	120.04 (19)	C10B—C9B—Cl2B	119.12 (15)
C3A—C2A—H2A	121.2 (10)	C8B—C9B—Cl2B	117.08 (15)
C1A—C2A—H2A	118.8 (10)	C9B—C8B—C7B	118.75 (18)
C2A—C1A—C17A	119.86 (19)	C9B—C8B—H5B	120.6 (10)
C2A—C1A—H1A	119.9 (10)	C7B—C8B—H5B	120.6 (10)
C17A—C1A—H1A	120.2 (10)	N11B—C12B—O2B	121.91 (17)
C13A—C17A—C1A	120.07 (18)	N11B—C12B—C17B	112.41 (16)
C13A—C17A—C12A	120.66 (17)	O2B—C12B—C17B	125.68 (17)
C1A—C17A—C12A	119.26 (17)	C12B—C17B—C13B	120.36 (17)
C17A—C12A—N11A	112.36 (16)	C12B—C17B—C1B	119.93 (17)
C17A—C12A—O2A	125.53 (18)	C13B—C17B—C1B	119.70 (18)
N11A—C12A—O2A	122.11 (17)	C17B—C13B—N5B	122.53 (17)
C12A—N11A—C14A	122.65 (16)	C17B—C13B—C4B	119.38 (18)
C12A—N11A—C16A	127.27 (16)	N5B—C13B—C4B	118.09 (17)
C14A—N11A—C16A	110.08 (15)	C13B—N5B—C14B	115.64 (16)
N11A—C16A—C15A	109.50 (16)	C13B—C4B—C3B	120.22 (19)
N11A—C16A—C10A	127.57 (18)	C13B—C4B—H4B	119.5 (10)
C15A—C16A—C10A	122.93 (18)	C3B—C4B—H4B	120.2 (10)
C16A—C10A—C9A	115.95 (18)	C4B—C3B—C2B	120.39 (19)
C16A—C10A—H6A	122.5 (10)	C4B—C3B—H3B	118.8 (10)
C9A—C10A—H6A	121.5 (10)	C2B—C3B—H3B	120.8 (10)
C10A—C9A—C8A	123.45 (18)	C3B—C2B—C1B	120.12 (19)
C10A—C9A—Cl2A	118.47 (15)	C3B—C2B—H2B	120.2 (10)
C8A—C9A—Cl2A	118.07 (15)	C1B—C2B—H2B	119.6 (10)
C9A—C8A—C7A	118.51 (17)	C17B—C1B—C2B	120.18 (18)
C9A—C8A—H5A	120.8 (10)	C17B—C1B—H1B	120.2 (10)
C7A—C8A—H5A	120.6 (10)	C2B—C1B—H1B	119.6 (10)

### Hydrogen-bond geometry (Å, °)

**Table:**

*D*—H···*A*	*D*—H	H···*A*	*D*···*A*	*D*—H···*A*
C1B—H1B···Cl1A^i^	0.94 (2)	2.73 (2)	3.637 (2)	162 (1)
C2A—H2A···O1B^ii^	0.93 (2)	2.54 (2)	3.264 (3)	135 (1)
C4B—H4B···N5A^iii^	0.94 (2)	2.56 (2)	3.422 (3)	154 (1)
C10A—H6A···Cl2B^iv^	0.94 (2)	2.67 (2)	3.585 (2)	165 (1)

Symmetry codes: (i) −*x*, −*y*+1, −*z*; (ii) −*x*+2, −*y*, −*z*; (iii) *x*−1, *y*, *z*; (iv) *x*, *y*−1, *z*−1.
